# Short-term effects of carbohydrates differing in glycemic index (GI) consumed at lunch on children’s cognitive function in a randomized crossover study

**DOI:** 10.1038/s41430-020-0600-0

**Published:** 2020-03-12

**Authors:** Kathrin Jansen, Jana Tempes, Alina Drozdowska, Maike Gutmann, Michael Falkenstein, Anette E. Buyken, Lars Libuda, Henrik Rudolf, Thomas Lücke, Mathilde Kersting

**Affiliations:** 1 Research Department of Child Nutrition, University Children’s Hospital, Ruhr University, Bochum, Germany; 2 University of Education, Freiburg, Germany; 3 Maria-Montessori-Allee 10, 53229 Bonn, Germany; 4 Institute for Work, Learning and Ageing (ALA), Bochum, Germany; 5 Public Health Nutrition, Institute of Nutrition, Consumption and Health, Paderborn University, Paderborn, Germany; 6 Department of Child and Adolescent Psychiatry, University Hospital Essen, University of Duisburg-Essen, Essen, Germany; 7 Department of Medical Informatics, Biometry and Epidemiology, Ruhr-University Bochum, Bochum, Germany

**Keywords:** Cognitive neuroscience, Paediatrics

## Abstract

**Background:**

Intervention studies suggest an influence of breakfast dietary glycemic index (GI) on children’s cognition. The Cognition Intervention Study Dortmund-GI-I study examined whether lunch dietary GI might have short-term effects on selected cognitive parameters.

**Methods:**

A randomized crossover study was performed at a comprehensive school on 2 test days. One hundred and eighty-nine participants (5th and 6th grade) were randomly assigned to one of the two sequences, medium-high GI (m-hGI) or high-medium GI (h-mGI), following block randomization. In the first period, one group received a dish containing hGI rice (GI: 86) ad libitum, the other mGI rice (GI: 62)—1 week later, in the second period, vice versa. Tonic alertness, task switching, and working memory updating were tested with a computerized test battery 45 min after beginning of lunch break. Treatment effects were estimated using the *t* test for normally distributed data or the Wilcoxon rank-sum test for non-normally distributed data.

**Results:**

The crossover approach revealed no effects of lunch dietary GI on the tested cognitive parameters in the early afternoon. However, we determined carryover effects for two parameters, and therefore analyzed only data of the first period. The reaction time of the two-back task (working memory updating) was faster (*p* = 0.001) and the count of commission errors in the alertness task was lower (*p* = 0.04) in the hGI group.

**Conclusion:**

No evidence of short-term effects of lunch dietary GI on cognition of schoolchildren was found. Potential positive effects on single parameters of working memory updating and tonic alertness favoring hGI rice need to be verified.

## Introduction

Children attending all-day schools are particularly challenged to maintain cognitive performance until the afternoon. A proper lunch might help to achieve this. However, it remains to be established which lunch composition is most suitable.

Glucose is the main energy source of the brain and its continuous supply is crucial [1]. Although glucose has short-term positive effects on cognitive performance [2, 3], high consumption of carbohydrates leading to fast and high increases in postprandial blood glucose might restrain cognitive potentials [4]. Instead, carbohydrate-rich foods causing a slower and prolonged rise of blood glucose were shown to improve attention and memory compared with carbohydrate-rich foods causing postprandial peaks [5–7]. The glycemic index (GI) ranks available carbohydrates provided by carbohydrate-rich foods by their effects on postprandial blood glucose concentrations. Consumption of breakfast with low-dietary GI resulted in better cognitive function in children than skipping breakfast or consuming breakfast with high-dietary GI [8–10]. However, a repeated-measures study did not report effects on cognition by breakfasts differing in their dietary GI [11]. Possible explanations are inconsistent methodologies and confounding factors of the studies [12]. To the best of our knowledge, studies on lunch dietary GI and short-term cognitive effects in children are not available up to now.

Within the Cognition Intervention Study Dortmund (CogniDo) series, we observed no negative short-term effect of having or skipping lunch on the cognitive performance of schoolchildren, contrasting to studies among adults showing postprandial fatigue after having lunch [13–17]. In fact, for working memory updating, having lunch might be temporarily beneficial for children [16]. Since lunch composition was not considered in these interventions, the aim of this study was to elucidate short-term effects of lunch dietary GI on the cognitive performance of schoolchildren.

## Methods

### Study design and participants

In accordance with previous CogniDo studies [15–17], this study was designed as a randomized, single blind 2 × 2 crossover intervention. The all-day “Comprehensive School Berger Feld” in Gelsenkirchen, Germany was chosen as venue, for a duration of 10 weeks (April–mid-June 2016).

Participants were recruited from the 5th and 6th grade (in total 12 classes). The students from the 6th grade had already participated in the previous CogniDo study (as 5th grade students). Children with a metabolic disorder, epilepsy or on a special diet were excluded. Children diagnosed with a learning disorder or insufficient knowledge of the German language (reported by the class teacher) were excluded post hoc from the analyses.

Each study arm consisted of a sequence of two treatments. In one arm, participants received a lunch with medium GI (mGI) rice in period 1 and 1 week later, in period 2, a lunch with high GI (hGI) rice (sequence m-hGI). In the other arm, the sequence was reversed (sequence h-mGI). Participants were randomly assigned to one of the two sequences following block randomization per class. Block sizes ranged from two to four participants. For allocation, a computer-generated list of random numbers was used. The 2 test days per individual were scheduled on the same weekday 1 week apart (except one class with 3 weeks in between due to lesson cancellation at short notice).

Lunch consisted of rice with ground beef sauce. Beforehand, commonly available rice and pasta products were tested by our staff for sensory properties and with regard to the acceptance by children. Hereupon, the GI of the four most promising products (two types pasta and rice, respectively) were analyzed by Sidney University’s Glycemic Index Research Service (SUGiRS), a certified laboratory for GI testing (ISO 26642:2010). Briefly, ten healthy adult volunteers received glucose and four test foods containing 50 g of digestible carbohydrates on different occasions after overnight fasting. Capillary blood samples were obtained for glucose measurements before and at regular intervals during 2 h after ingestion. Using the incremental area under each 2-h plasma glucose response curve (iAUC), GI values for each test food relative to the reference were calculated. Since the two rice types (62 vs. 86) differed more in their GI than the two pasta types (48 vs. 54), rice was chosen for intervention.

### Study schedule

**Fig. 1:**
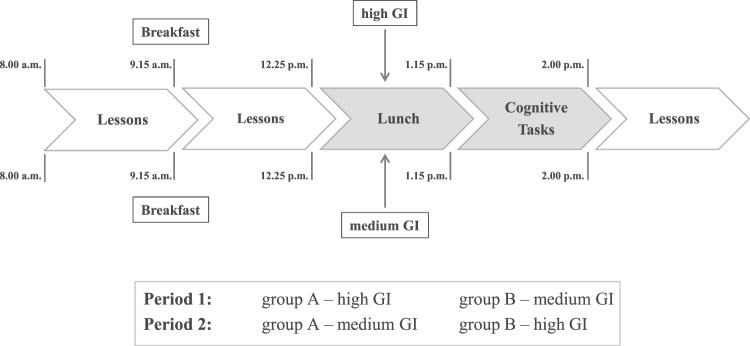
Study schedule of the cross over study. Each test day started at 9:15 a.m. with a standardized breakfast; on the first test day at 12:25 p.m. participants from group A received high GI rice, group Breceived medium GI rice (period 1); on the second test day vice versa (period 2); cognitive assessment was performed between 1:15 p.m. and 2 p.m.

The study design was integrated in the regular school routine. Each test day started at 09:15 a.m. with a standardized breakfast for each participant (bread from wholemeal flour, margarine, salami or Gouda cheese, and carrot sticks) ad libitum. At 12:25 p.m., the start of the regular lunch break, subjects received either lunch with mGI or hGI rice and ground beef sauce (Fig. 1). The amount of consumed rice and sauce was individually assessed by weighing plates before and after the meal. The estimated meal glycemic load (GL) of the consumed rice portion was obtained by multiplying the amount of rice-carbohydrates consumed by the GI of the respective rice (GL = GI × carbohydrate content (g) per portion/100). Carbohydrates provided by the sauce were ignored. Water was available at any time. After finishing lunch and a short break, cognitive assessment started at 1:15 p.m. in the school’s computer room.

Between the morning and the lunch break participants were asked to refrain from eating and drinking (except for water/unsweetened tea). The children were supervised during the breaks by the study staff. In addition, the participants completed a questionnaire on their food and beverage consumption on each intervention day.

### Cognitive assessment

Cognitive functions were assessed using a computerized test battery consisting of three tasks designed by the Institute for Work, Learning and Ageing (ALA Institute) in Bochum, Germany as described before [17]. Briefly, each task was explained by the study personal and the participants had the chance to practice once in advance in a training mode. After a 5 min break with low-physical activity, the actual cognitive testing started. Subjects were asked to respond as quickly as possible without neglecting accuracy. The cognition tasks were applied in consistent order: task switching, working memory updating, and tonic alertness.

#### Task switching

**Fig. 2:**
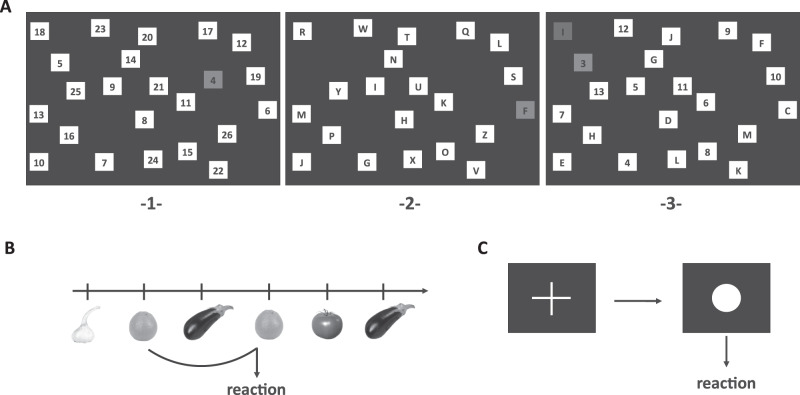
Computerized cognitive tasks. **a** Visual attention and task switching measured by switch task. The task comprised of three sections. **1** First section, numbers (nonswitch). Numbers had to be clicks in ascending order with the mouse curser. **2** Second section, letters (nonswitch). Letters from A to Z had to be clicked alphabetically. **3** Third section, number and letters (switch). Numbers and letters had to be clicked alternately in ascending order (i.e., 1-A-2-B-3-C…). **b** Working memory updating measured by two-back task. Fruits and vegetables were displayed on a computer screen. A predefined key had to be pressed when the current image was the same as the image two trials back. **c** Tonic alertness measured by alertness task. A predefined key had to be pressed as soon as a white circle appeared on the screen. Appearance of a white cross required no reaction.

Visual attention and task switching were measured using an alternative version of the Trail Making Task in three sections: the first two sections (numbers and letters) in a nonswitch condition, and the third section (letters and numbers) in a switch condition (Fig. 2a).

In the first section, black numbers from 1 to 26 in white squares were presented in an irregular order on a computer screen (Fig. 2a-1). Numbers had to be clicked in ascending order. When numbers were clicked correctly the squares turned green, otherwise red. Correctly processed squares faded out. The maximum time to finish was 3 min.

The second section used letters from A to Z instead of numbers (Fig. 2a-2).

**Fig. 3:**
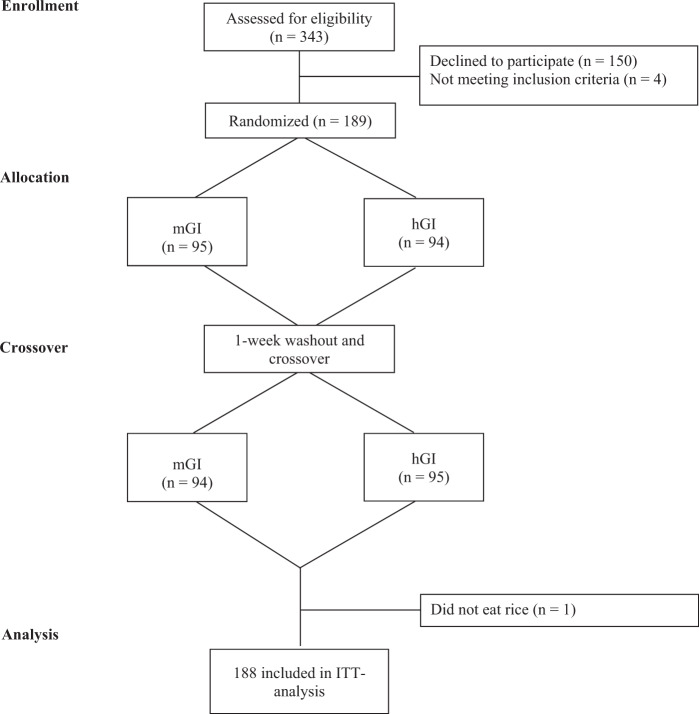
Flow diagram for crossover study. Intention-to-treat analysis (ITT). mGI medium glycemic index, hGI high glycemic index.

In the final section, the 26 squares contained numbers from 1 to 13 and letters from A to M (Fig. 2a-3). Participants had to alternately click numbers and letters in ascending order (i.e. 1-A-2-B-3-C). Outcomes included total reaction time (RT) for numbers (items 2–26), total RT for letters (items 2–13), and switch costs, i.e. the processing time of the third section minus the first section minus the difference between the first 12 items of the second section and the first 12 items of the first section. Negative switch costs were regarded as implausible and excluded.

#### Working memory updating (two-back task)

To assess capability in holding and manipulating information for a short time, the *n*-back task was used in a two-back condition. Subjects were asked to monitor a sequence of 106 consecutive pictures of fruits and vegetables presented in the middle of the screen (Fig. 2b). When the current picture matched the picture presented two trials earlier (*n* − 2), participants had to press a key. The stimuli were presented for 500 ms (interstimulus interval: 2100 ms, maximal RT: 1400 ms). No feedback was given. Twenty-one pictures were targets (same picture as two trials before).

The outcome variables were: ratio of missings (no reaction while reaction was required), ratio of false alarms (reaction while no reaction was required), and mean RT while reaction was required.

#### Tonic alertness

A simple reaction task was used to measure tonic alertness. A white fixation cross was presented on a black screen (Fig. 2c). In a response stimulus interval of 3300 ms (±20%), a circle followed the cross and the subjects had to press a button as soon as the circle appeared (maximal RT 1500 ms). The test included 50 items. The outcome variables were the mean RT (ms), the deviation of RT (ms), the number of omission errors (no reaction after 1500 ms), and the number of commission errors (reaction during the presence of the fixation cross).

### Statistical analyses

All analyses were performed using the statistical software package IBM® SPSS® Statistics for Windows, version 25.0 (IBM Corp., Armonk, NY, USA). *p* < 0.05 was considered statistically significant.

The parameters of the cognitive tasks were used as outcome variables, all interval-scaled. Statistical analysis was performed in accordance with the Consolidated Standards of Reporting Trials statement and Wellek and Blettner [18, 19]. The sums of the two individual values of the outcome variables of period 1 and 2 were compared between both arms using an unpaired *t* test for normally distributed data and the Wilcoxon rank-sum test for non-normally distributed data to examine potential carryover effects. If no carryover effects were observed, results from both days were considered for the treatment effect. Briefly, individual differences of the particular outcomes of both test days (test day 1–test day 2) were compared between both sequences (hGI–mGI vs. mGI–hGI). In case of carryover effects, only results from day 1 were considered.

Associations of GI with cognitive parameters were adjusted for GL using a linear mixed model. In addition, period effects were determined with this model. GL, period, and GI were treated as fixed effects, subjects as random.

## Results

### Participants

Out of 343 students in the 5th and 6th grade, 193 students (56%) with informed written consent participated. Of these, four were excluded due to diagnosed learning disorder, one student did not eat lunch on one of the test days (Fig. 3). Thus, intention-to-treat analysis was performed using cognitive performance data of 188 subjects. Characteristics of the 188 included participants are shown in Table 1.

**Table 1:** Characteristics of the study population, glycemic load, and meal consumption.

	Medium-high GI (*n* = 95)			High-medium GI (*n* = 94)		
	Period 1	Period 2	*p*	Period 1	Period 2	*p*
Age (years), mean ± SD	11.8 ± 0.7			11.7 ± 0.8		
Female, *n* (%)	43 (45)			41 (44)		
Glycemic load	68 ± 34	103 ± 51	<0.001	111 ± 62	66 ± 37	<0.001
Meal consumption (g)	266 ± 147	264 ± 133	0.89	292 ± 162	270 ± 155	0.08

Sequence medium-high GI: participants received lunch with medium GI rice in the first period and high GI rice in the second period; sequence high-medium GI: vice versa, paired *t* test, mean ± SD, *p* < 0.05.

*GI* glycemic index.

### Lunch dietary GI and cognition

Statistical analyses revealed no significant differences between lunch based on medium or high GI rice for most parameters of the selected cognitive outcomes (Table 2).

**Table 2:** Effects of lunch with medium and high GI rice on cognitive performance in schoolchildren.

	Medium-high GI						High-medium GI						
	Period 1			Period 2			Period 1			Period 2			GI
	$\tilde x$	25th	75th	$\tilde x$	25th	75th	$\tilde x$	25th	75th	$\tilde x$	25th	75th	*p*
Switch (*n* = 170)													
Switch costs [s]	**26.6**	16.3	35.2	**19.7**	10.5	31.2	**25.2**	14.9	36.7	**20.0**	11.4	28.2	0.68*
Visual search letters [s]^‡^	**30.0**	26.2	35.8	**30.0**	25.3	36.6	**29.2**	25.4	37.0	**29.5**	25.5	36.7	0.14
Visual search numbers [s]	**45.7**	40.2	52.9	**48.4**	41.8	56.9	**45.9**	40.3	51.8	**49.7**	43.1	56.8	0.54*
2-back (*n* = 188)													
RT [ms]	**559.5**	497.7	641.8	**535.2**	459.1	608.0	**533.6**	426.1	604.4	**488.5**	421.1	572.4	0.001^a^
Ratio of missings (%)	**33.3**	19.0	42.9	**33.3**	19.0	47.6	**28.6**	19.0	42.9	**33.3**	19.0	47.6	0.92*
Ratio of false alarms (%)	**7.1**	3.5	15.3	**5.9**	2.4	11.8	**7.1**	2.4	17.6	**5.9**	1.2	13.5	0.41^#,^*
Alertness (*n* = 188)													
Mean RT [ms]	**332.5**	303.5	369.9	**356.6**	314.2	400.8	**330.9**	296.6	382.3	**357.1**	310.2	391.9	0.42*
Deviation of RT [ms]	**112.2**	80.8	155.9	**135.0**	98.6	179.5	**100.6**	76.1	151.1	**125.0**	90.2	176.6	0.94^#,^*
Count of omission errors (*n*)	**0**	0	1	**0**	0	1	**0**	0	1	**0**	0	1	0.24^#^
Count of commission errors (*n*)	**2**	1	4	**2**	1	4	**1**	1	3	**1**	0	3	0.04^#,a^

Medium-high GI: participants received lunch with medium GI rice in the first period and high GI rice in the second period; high-medium GI: vice versa.

*GI* glycemic index, *GL* glycemic load, *RT* reaction time.

^‡^First 12 reactions; switch costs = (mean RT switch task) − (mean RT number task) − (mean RT first 12 reactions of letter task − mean RT first 12 reactions of number task). ${\tilde x}$ = Median (presented in bold), 25th and 75th percentiles; two sample *t* test.

^#^Wilcoxon’s ranked sum test.

*Period effects detected using linear mixed model.

^a^*p* values for RT (two back) and count of commission errors (alertness) were only calculated for period 1.

For one outcome of the two-back task (RT) and one of the alertness task (count of commission error) carryover effects were detected. Thus, statistical analysis was only performed for period 1. Eating lunch with hGI rice resulted in shorter RT compared with lunch with mGI rice. Similarly, number of errors of the alertness task were lower after lunch with hGI rice.

There were period effects for some parameters: switch costs improved in period 2 compared with period 1 in both groups while the RT for visual search numbers slowed down. Within the two-back task and the alertness task, RT and the ratio of false alarms decreased in both groups in period 2 while the ratio of missings increased.

### Effect adjustment for estimated lunch GL

Estimated meal GL differed significantly between both periods, while the amount of lunch consumed did not differ (Table 1). Including the estimated meal GL as covariate in the analysis revealed no significant association with cognitive parameters for either GI or GL (Table 3).

**Table 3:** GL-adjusted GI effects on cognitive parameters.

	Medium GI		High GI		
	Estimate	95% confidence interval	Estimate	95% confidence interval	*p*
Switch (*n* = 170)					
Switch costs [s]	24.6	21.9, 27.3	25.9	23.1, 28.6	0.49
Visual search letters [s]^a^	32.3	30.7, 33.8	32.3	30.7, 33.8	0.99
Visual search numbers [s]	49.1	47.3, 50.8	48.3	46.6, 50.0	0.32
2-back (*n* = 188)					
RT [ms]	540.8	524.0, 557.7	528.8	511.9, 545.6	0.17
Ratio of missings (%)	33.9	31.2, 36.6	34.6	31.9, 37.3	0.68
Ratio of false alarms (%)	12.6	10.2, 14.9	11.8	9.4, 14.2	0.38
Alertness (*n* = 188)					
Mean RT [ms]	352.6	341.3, 364.0	358.8	347.5, 370.2	0.23
Deviation of RT [ms]	142.8	129.1, 156.5	139.3	125.6, 153.1	0.69
Count of omission errors (*n*)	1.2	0.6, 1.8	1.4	0.7, 2.0	0.57
Count of commission errors (*n*)	3.0	2.3, 3.7	2.9	2.2, 3.6	0.87

*GI* glycemic index, *GL* glycemic load, *RT* reaction time.

^a^First 12 reactions; switch costs = (mean RT switch task) − (mean RT number task) − (mean RT first 12 reactions of letter task) − mean RT first 12 reactions of number task; analyzed with linear mixed model.

Additional analyses for dietary GI and GL effects were performed considering only participants who had fully adhered to the study protocol (no eating and drinking except for lunch and water/unsweetened tea). The outcomes were not different from the results described above (data not shown).

## Discussion

In our previous CogniDo studies, lunch caused no decline in cognitive performance, even indicating small positive effects [15, 16]. In the present study, we investigated for the first time short-term cognitive effects of carbohydrate-rich lunch differing in GI. Our results indicate small but significant effects favoring hGI rice with regard to two of the tested cognition parameters: working memory updating (two-back task: RT) and alertness (alertness task: count of commission errors), respectively, although only results from period 1 could be considered for this analysis due to carryover effects. A carryover effect is defined as the effect of the treatment from the first period on the response at the second period. Accordingly, rice GI would have a lasting impact on cognition for over a week. Considering, that food digestion takes ~6–8 h and the fact that numerous different carbohydrate sources were consumed by the children during the week between the two test periods, a carryover effect in the original sense appears rather implausible. Thus, our results suggesting that hGI rice would improve these two parameters of cognitive performance in period 1 should be interpreted cautiously. It may be possible that the cognitive parameters of the two groups differed from the start, independent of the consumed rice. However, cognitive improvements by a dish based on a high GI food are in line with findings from Micha et al. showing an improved short-term memory and vigilance in 11–14-year-old children after eating a hGI breakfast [20, 21]. On the contrary, Cooper et al. showed that a low-GI breakfast (GI = 48) improves the response time and accuracy of working memory and attention in 12-year-old children across the school morning as compared with hGI breakfast (GI = 72) and breakfast omission, probably by causing lower peak blood glucose concentrations [8]. Similarly, hGI breakfast has been associated with a significant decline in accuracy of attention compared with low-GI breakfast [9].

Apart from the differences, we found for the two cognitive parameters in period 1, none of the other parameters were affected by lunch composition within this crossover study. Some studies investigating the influence of GI in breakfast found positive cognitive effects for either low or hGI. This inconsistency compared with our study could be due to the prolonged fasting before breakfast. In our study, all participants were offered a standardized breakfast to ensure the same conditions for the cognition tasks. Consequently, the fasting phase took merely 3 h. Blood glucose concentrations usually start to increase 15 min post prandial, reaching peak concentrations after ~30–60 min and return to baseline (or below) within 3–4 h. In addition, even though breakfast was standardized with regard to the food ingredients, the amount of breakfast consumed was not controlled. This might have influenced the outcome after lunch. Moreover, it has to be considered that rice was served with ground beef sauce, which makes the prediction of the glycemic response difficult. Principally, the simultaneous intake of rice with meat and fat leads to a prolonged digestion and a reduced glycemic response [1, 22]. Thus, blood glucose concentrations may not have differed sufficiently between our intervention conditions. Measuring glucose responses with continuous glucose monitoring would have been helpful to differentiate between both test meals but was impossible in healthy children.

The timeframe between eating and cognitive testing might be of importance as well. Others reported significant effects of a low-GI breakfast 2–3 h after ingestion on the grounds of lower but prolonged blood glucose concentrations that prevent a decline in cognitive performance later in the morning [6, 8, 9]. The timeframe in our study was only 45 min. Possibly, differences would have been visible later in the afternoon.

In addition to the GI, we considered meal sizes and estimated the GL of the consumed rice portion. Although GL differed significantly between groups, adjusting the effects of dietary GI for GL showed no association with the cognitive performance. This is in line with findings from Brindal et al. reporting no changes in speed of processing, working memory, short-term memory, perceptual speed, and inspection time over a time course of 3 h in 10–12-year-old children after eating breakfasts with different GLs [23]. On the contrary, high-GL breakfasts have been shown to improve working memory and speed of information processing 90 min after breakfast [20], further highlighting the discrepancies between the studies investigating the effect of GI and GL on cognition.

Finally, it must be mentioned that we discovered period effects within our crossover design. Independent of the rice GI, children showed either improved or impaired results for some cognitive parameters on the second test day, may be due to learning effects. Learning effects appear independently of carryover effects, which in turn would be a metabolically based cognitive consequence of the dietary intervention lasting until the second cognitive test. On the other hand, it is quite challenging to motivate children throughout the testing, especially when a task proceeds rather monotonously.

A strength of the present study is that it was not performed under clinical conditions but tested schoolchildren in their everyday school environment. Thus, our results indicate effects of carbohydrates differing in GI consumed at lunch under real-life conditions. Considering large interindividual differences in cognition, the crossover design is another strength of our study, as every subject acts as his/her own control. In addition, the GI of the rice used for our dietary intervention was assessed in a certified lab according to ISO standards.

However, our approach under real-life conditions is vulnerable to confounding factors. Schoolchildren and their peers tend to distract or influence each other when a whole class is tested simultaneously. Although, the children were supervised during the cognitive testing to assure they stay focused, this could be one explanation for the deterioration in some cognitive parameters. Another limitation is that the GI difference between the rice types may not have been sufficient. Since our aim was to examine our question within an everyday school life, we chose a lunch from which we know that children like it. Measuring glucose responses with continuous glucose monitoring was not possible.

In conclusion, the present study provides no clear evidence that the dietary GI of lunch influences the short-term cognitive performance of schoolchildren 45 min after beginning of the lunch break. Potential beneficial effects of lunch based on hGI rice on working memory updating and alertness warrant attention. It is also of interest whether extending time between lunch and testing might influence the cognitive performance.
