# Correction: Short-term effects of carbohydrates differing in glycemic index (GI) consumed at lunch on children’s cognitive function in a randomized crossover study

**DOI:** 10.1038/s41430-021-01046-6

**Published:** 2021-11-26

**Authors:** Kathrin Jansen, Jana Tempes, Alina Drozdowska, Maike Gutmann, Michael Falkenstein, Anette E. Buyken, Lars Libuda, Henrik Rudolf, Thomas Lücke, Mathilde Kersting

**Affiliations:** 1 Research Department of Child Nutrition, University Children’s Hospital, Ruhr University, Bochum, Germany; 2 University of Education, Freiburg, Germany; 3 Maria-Montessori-Allee 10, 53229 Bonn, Germany; 4 Institute for Work, Learning and Ageing (ALA), Bochum, Germany; 5 Public Health Nutrition, Institute of Nutrition, Consumption and Health, Paderborn University, Paderborn, Germany; 6 Department of Child and Adolescent Psychiatry, University Hospital Essen, University of Duisburg-Essen, Essen, Germany; 7 Department of Medical Informatics, Biometry and Epidemiology, Ruhr-University Bochum, Bochum, Germany

**Keywords:** Cognitive neuroscience, Paediatrics

Correction to: *European Journal of Clinical Nutrition* 10.1038/s41430-020-0600-0

The article Short-term effects of carbohydrates differing in glycemic index (GI) consumed at lunch on children’s cognitive function in a randomized crossover study, written by Kathrin Jansen, Jana Tempes, Alina Drozdowska, Maike Gutmann, Michael Falkenstein, Anette E. Buyken, Lars Libuda, Henrik Rudolf, Thomas Lücke, Mathilde Kersting was originally published Online First without Open Access. After publication in volume 74, issue 5, page 757 - 764 the author decided to opt for Open Choice and to make the article an Open Access publication. Therefore, the copyright of the article has been changed to © The Author(s) 2020 and the article is forthwith distributed under the terms of the Creative Commons Attribution 4.0 International License, which permits use, sharing, adaptation, distribution and reproduction in any medium or format, as long as you give appropriate credit to the original author(s) and the source, provide a link to the Creative Commons licence, and indicate if changes were made.

The images or other third party material in this article are included in the article’s Creative Commons licence, unless indicated otherwise in a credit line to the material. If material is not included in the article’s Creative Commons licence and your intended use is not permitted by statutory regulation or exceeds the permitted use, you will need to obtain permission directly from the copyright holder.

To view a copy of this licence, visit http://creativecommons.org/licenses/by/4.0/.

